# Interactions among Low Dose of Methotrexate and Drugs Used in the Treatment of Rheumatoid Arthritis

**DOI:** 10.1155/2013/313858

**Published:** 2013-05-12

**Authors:** Marinella Patanè, Miriam Ciriaco, Serafina Chimirri, Francesco Ursini, Saverio Naty, Rosa Daniela Grembiale, Luca Gallelli, Giovambattista De Sarro, Emilio Russo

**Affiliations:** ^1^Department of Health Science, School of Medicine, University of Catanzaro, Viale Europa, 88100 Catanzaro, Italy; ^2^Pharmacovigilance's Center Regione Calabria, University Hospital Mater Domini, 88100 Catanzaro, Italy; ^3^Rheumatology Research Unit and Ph.D. Program in Molecular Oncology, Experimental Immunology and Development of Innovative Therapies, University of Catanzaro, 88100 Catanzaro, Italy

## Abstract

Methotrexate (MTX) is a nonbiological disease-modifying antirheumatic drug that has shown both a good control of clinical disease and a good safety. Usually drug-drug interactions (DDIs) represent the most limiting factor during the clinical management of any disease, in particular when several drugs are coadministered to treat the same disease. In this paper, we report the interactions among MTX and the other drugs commonly used in the management of rheumatoid arthritis. Using Medline, PubMed, Embase, Cochrane libraries, and Reference lists, we searched for the articles published until June 30, 2012, and we reported the most common DDIs between MTX and antirheumatic drugs. In particular, clinically relevant DDIs have been described during the treatment with MTX and NSAIDs, for example, diclofenac, indomethacin, or COX-2 inhibitors, and between MTX and prednisone or immunosuppressant drugs (e.g., leflunomide and cyclosporine). Finally, an increase in the risk of infections has been recorded during the combination treatment with MTX plus antitumor necrosis factor-**α** agents. In conclusion, during the treatment with MTX, DDIs play an important role in both the development of ADRs and therapeutic failure.

## 1. Introduction

Rheumatoid arthritis (RA) is a chronic and autoimmune disease affecting about 1% of people, with the highest incidence between 40 and 70 years [1]. Drugs able to reduce inflammation and cells activation may be used in the management of RA. In particular, nonsteroidal anti-inflammatory drugs (NSAIDs) as well as immunosuppressive agents (i.e., glucocorticoids), disease-modifying antirheumatic drugs (DMARDs), and agents that are able to block the proinflammatory cytokine tumor necrosis factor-*α* (anti-TNF-*α*) may be used (Table 1).

NSAIDs, acting on cyclooxygenases, are able to control the inflammation and the clinical symptoms [2–4], but not the disease's progression; their use as monotherapy for a long time is limited for the development of adverse drug reactions (ADRs) [5].

Glucocorticoids (e.g., dexamethasone and prednisone) are anti-inflammatory and immune suppressor agents that are able to reduce the inflammation and the progression of RA, through the inhibition of cytokines secretion and osteoclasts activation [6–15].

However, even if they represent a first-line treatment in patients with RA, their use is limited for the development of serious ADRs such as loss of bone mass, increased risk of fractures, infections, diabetes and hypertension [16–18]. The DMARDs group, includes both nonbiological and biological drugs; between the DMARDs, methotrexate (MTX) due to its good safety and clinical efficacy, represents the first choice for the treatment of RA [19]. When a 6-month treatment with conventional drugs (NSAIDs, glucocorticoid, and DMARDs) is ineffective or when RA is severe (e.g., early destruction or unfavorable prognosis), the treatment with biological DMARDs may be started. A combination treatment between MTX and glucocorticoids or biological drugs is also often considered (Table 2) [20–22]. In patients with RA, the Food and Drug Administration (FDA) approved the use of anti-TNF-*α* (infliximab, adalimumab, golimumab, certolizumab, pegol, and etanercept) as combined therapies [23]. Previously, we documented that a multidrug treatment may be related to a higher development of ADRs and/or drug-drug interactions (DDIs) [24–42], and this represents a major issue during the clinical management of any disease requiring polytherapy. Based on this background, the aim of the present review is to describe the DDIs between MTX and the other drugs used in the treatment of patients with RA.

## 2. Methods

The Medline, PubMed, Embase, and Cochrane library databases were searched for articles published until June 30, 2012. Secondary search included articles cited in reference lists identified by the primary search. Records were first screened by title/abstract before full text articles were retrieved for eligibility evaluation. Remaining articles were then subjected to a citation search before a final hand search of all reference lists. Papers were deemed eligible if they included any form of words: “rheumatoid arthritis,” “antirheumatic drugs,” “NSAIDs,” “glucocorticoids,” “DMARDs,” “biologic drugs,” and “drug-drug interaction.” All citations were downloaded into Endnote software version 14, and duplicates were deleted. MP, MC, and FU screened all articles by title/abstract to determine their eligibility, and LG reviewed a random sample of 15% in order to evaluate the reliability of the selection process. In order to avoid a bias of exclusion, the full text articles were retrieved following first round exclusions and were also subjected to two independent eligibility reviews (MP 100% and LG 15%), this time with perfect agreement. The studies evaluated as eligible were enclosed in the present review.

## 3. Methotrexate Pharmacology

### 3.1. Pharmacokinetic

After oral administration, MTX is rapidly adsorbed, with a Tmax of 2 hours and a terminal half-life of 8–10 hours. In plasma, MTX binds reversibly to albumin (60–70%) and rapidly accumulates inside red blood cells through the reduced folate carrier. In red cells, MTX receives 2–5 glutamate moieties and is retained as MTX polyglutamates (MTXGlu) [43, 44]. The enzyme gamma-glutamyl hydrolase removes the terminal glutamate molecules; therefore, MTX returns to its monoglutamate form and can be transported out of the red cells by multidrug-resistant protein [43]. MTX is predominantly eliminated as an unmodified drug by the kidney (80%) via the human organic anion transporter-3 (HTO-3) in the renal proximal tubule.

### 3.2. Pharmacodynamic

**Figure 1:**
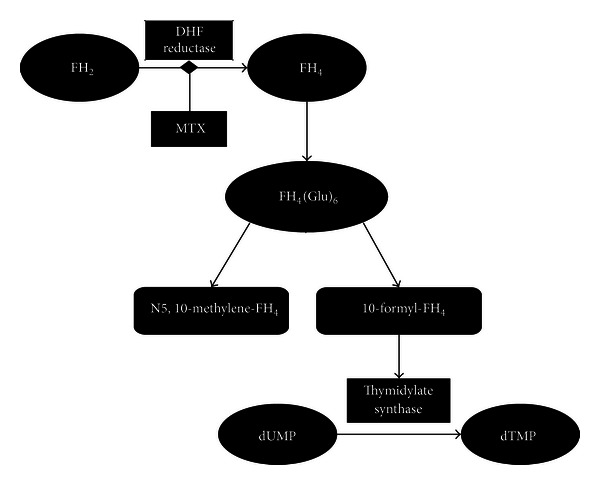
Schematic representation of methotrexate's pharmacodynamic. Methotrexate (MTX) indirectly blocks the formation of dTMP by inhibiting DHF reductase. FH_2_, which accumulates following the inhibition of DHF reductase, in turn inhibits thymidylate synthase. DHF = dihydrofolate; FH_2_ = dihydrofolic acid; FH_4_ = tetrahydrofolic acid; dTMP = thymidine monophosphate; and dUMP = deoxyuridine monophosphate.

MTX is an “antimetabolite” that is able to block the chain elongation of DNA leading to the arrest of cell cycle. Folic acid analogues, competitively and reversibly inhibit the enzyme dihydrofolate reductase (DHF reductase) that catalyzes the transformation of dihydrofolic acid (FH_2_) into tetrahydrofolic acid (FH_4_) (Figure 1).

FH_4_, inside the cells, undergoes the addition of 6 glutamic-acid residues, by the enzyme folic-glutamate synthase to obtain FH_4_(Glu)_6_. Then, it is transformed into two molecules: N5,10-methenyl-FH_4_ and 10-formyl-FH_4_ (Figure 1).

N5,10-methenyl-FH_4_ acts as a donor of monocarboniose units in the reaction that forms thymidine monophosphate (dTMP) from deoxyuridine monophosphate (dUMP); this reaction is catalyzed by the enzyme thymidylate synthase.

Thymidine is required for the synthesis of DNA and RNA. MTX indirectly blocks the formation of dTMP by inhibiting DHF reductase. Furthermore, FH_2_, which accumulates following the inhibition of DHF reductase, in turn inhibits thymidylate synthase (Figure 1). Through this mechanism, MTX blocks the synthesis of DNA and RNA and acts as an antimetabolite, mainly during the S-phase of cell cycle, by presenting a greater cytotoxic effect in cells with high turnover, for example, cancer cells [22]. Moreover, it has been also reported that MTX is able to inhibit the production of cytokines (e.g., IL-1), leukotriene B4, and histamine [45, 46].

In this light, MTX is used to treat patients with hematological malignancies as well as patients with rheumatoid arthritis, juvenile rheumatoid arthritis, and skin disorders (e.g., psoriasis, dermatomyositis, systemic sclerosis, morphoea, cutaneous sarcoidosis, and different types of eczema).

## 4. Methotrexate's Interactions with Drugs Commonly Used in RA Treatment

DDIs represent the commonest causes of ADRs, particularly in the elderly and can be classified into two main groups pharmacokinetic or pharmacodynamic [52]. 

### 4.1. MTX and NSAIDS

A recent Cochrane systematic review by Colebatch and coworkers [53] evaluating 8621 studies documented that only 17 publications reported a concurrent use of MTX and NSAID, but none reported ADRs on lung, liver, or renal function and no increase in MTX withdrawal or in other major toxicity. On the other hand, a pharmacokinetic DDIs, on HOAT-3, has been described during the treatment with MTX plus NSAIDs, particularly with ketoprofen, indomethacin, naproxen, and diclofenac [47, 48]. Moreover, an experimental study has recently documented that etoricoxib is able to inhibit the HOAT-3 in a competitive and dose-dependent manner [49]; therefore, DDI could be hypothesized during the treatment with MTX. 

In contrast, up to date no data have been published concerning a DDI between celecoxib and MTX [50, 51] (Table 3).

### 4.2. MTX and Glucocorticoids

An experimental study demonstrated that an oral pretreatment with dexamethasone reduces up to 53% of the biliary clearance of MTX, inducing a hepatocellular impairment [65].

Although liver toxicity has been reported in children with brain tumor treated with MTX and dexamethasone [66], definitive data have not been published concerning possible DDIs between MTX and glucocorticoids.

In agreement, Malysheva et al. [67] failed to report the development of DDIs during the concomitant treatment with MTX plus prednisolone.

### 4.3. MTX and DMARDs

The combination of MTX and either leflunomide or sulphasalazine in nonresponders RA patients reduced pain symptoms but induced an increase in serious ADRs. In particular, since pharmacokinetic DDIs were not reported in patients treated with MTX (mean dose 17.2 mg per week) plus leflunomide (100 mg daily for 2 days as a loading dose followed by 10 to 20 mg daily) [68], several papers reported an increase in both liver toxicity and blood dyscrasia (i.e., pancytopenia) (Table 4). In this light, the UK manufacturer suggests that the coadministration of MTX and leflunomide is not advisable [54–59]. In agreement Lee et al. [60] reported an increased risk of liver fibrosis in patients with RA treated with leflunomide plus methotrexate. However, the Smile study evaluating 2975 patients with RA, recently documented the safety of the association between MTX and leflunomide [69]. In contrast, Katchamart et al. [61] evaluating 19 clinical trials (2025 patients) documented that the concurrent treatment with MTX plus sulphasalazine is correlated with an increase in both liver and gastrointestinal toxicity (Table 4). Finally, even if sulphasalazine displaces MTX from its plasmatic albumin site, no net change in free MTX concentration or an increased risk of toxicity was underlined. This is probably due to the percentage (50%) of binding and the increased displacement would likely lead to an increased elimination sulphasalazine is a HOAT-1 and HOAT-3 inhibitors; therefore, it is able to inhibit the renal excretion of MTX [62]. However, the clinical impact of this DDI should be better investigated in clinical trials.

### 4.4. MTX and Immunosuppressant

Usually, cyclosporine administered as monotherapy can induce kidney toxicity, so it should be started at lower dose (Table 4). It was previously suggested that combined treatment with cyclosporine and other antirheumatic drugs, such as MTX, is very promising [78]. However, an open-label pharmacokinetic study published by Baraldo et al. [79] documented in 26 RA patients treated for 14 days with MTX (7.5 to 22.5 mg weekly) plus cyclosporine (1.5 mg/kg bid) a significant increase (*P* < 0.01) in mean area under the curve (AUC) of MTX (26%), with a significant decrease (80%; *P* < 0.001) of its active metabolite (7-hydroxymethotrexate). This DDI is related to a lower activity and a higher toxicity. In agreement, more recently, another study reported an increase in kidney ADRs (e.g., hypertension or increase in serum creatinine) in RA patients treated with cyclosporine plus MTX in comparison to patients receiving cyclosporine alone [63]. In contrast, Sarzi-Puttini et al. [64] did not show any increase in ADRs in patients treated with cyclosporine (3 mg/kg) + MTX (from 7.5 to 10 mg weekly) in respect to patients treated with cyclosporine alone. At the moment, no DDIs during the treatment with MTX + mycophenolate mofetil have been reported; therefore, this coadministration seems to be well tolerated (Table 4).

### 4.5. MTX and Biological Drugs

The cotreatment with anti-TNF-*α* plus MTX induces synergistic effects on the immunosuppressive activity of MTX, even if this treatment may be related to an increased risk of infections [70–72]. However, no pharmacokinetic DDIs were documented during the treatment with etanercept (subcutaneous dose) plus MTX (oral dose) [72]. 

Similarly, several clinical trials performed in patients with active RA without an adequate response to MTX; the addition of adalimumab to MTX achieved an improvement of RA symptoms, without the development of pharmacokinetic DDIs [73, 80, 81].

However, this treatment may be related to an increase in liver enzymes; therefore, it is prudent to increase the frequency of liver enzyme monitoring in patients receiving this cotreatment. Other data documented the absence of pharmacokinetic DDIs during the contemporary administration of MTX plus rituximab [82], tocilizumab [74], certolizumab pegol [75], or golimumab [76] (Table 5).

Abatacept, a modulator of T-lymphocyte activation, approved for the treatment of active RA, has been recommended for nonresponder patients to DMARDs such as MTX or anti-TNF-*α* [77]. A recent Cochrane review demonstrated significant lower risk of serious ADRs during the treatment with abatacept in respect to other biological drugs [83]. Since an interaction between this drug and MTX has not been established, the ACR recommend the coadministration of abatacept and MTX; abatacept in monotherapy is not recommended due to the lack of data.

## 5. Discussion and Conclusion

The depletion of folate and the inability of the cells to synthesize DNA at the same time can explain the antineoplastic/antirheumatic effects possessed by MTX and also its toxicity. The suppressive effect of MTX on hematopoietic system and the risk of fibrosis, cirrhosis, or gastrointestinal ulcer require a frequent monitoring of functional parameters. The supplementation of folic acid is helpful to reduce the MTX's toxicity [84]. The importance of pharmacovigilance studies in patients treated with MTX is also related to risk of DDIs that may increase the MTX's toxicity. After absorption, MTX is bounded to the serum albumin for its distribution and therefore can be displaced by other drugs that are coadministered. The kidney clearance of MTX may be reduced by NSAIDs or DMARDs, while the risk of liver toxicity and bone marrow suppression is increased during the treatment with drugs with similar ADRs [84, 85]. In conclusion, during the treatment with MTX, it is necessary to keep in consideration the risk of DDIs that are able to increase the risk of hepatotoxicity, nephrotoxicity, myelotoxicity, and other toxic effects.

## 6. Summary

- DDIs are very important and must be timely recognized in order to prevent the development of ADRs or therapeutic failure.
- Low dose of MTX represents a safe treatment for patients with RA receiving other treatments with antirheumatic drugs, since some DDIs may occur.
- The association MTX + NSAIDs or etoricoxib may be related to an increase of MTX toxicity.
- The association MTX + leflunomide or MTX + sulphasalazine may induce liver toxicity and/or blood toxicity.
- Very few data have been published regarding the association between MTX and biological DMARDs.
- In order to prevent the development of DDIs, it is important to decrease the dosage of MTX or of the drugs that are coadministered.
- In the presence of ADRs during the treatment, it is useful to evaluate the plasma concentration of the drugs.

## Figures and Tables

**Table 1:** Drugs used in the management of rheumatoid arthritis.

Drugs for symptomatic control		DMARDs	
NSAID	Corticosteroids	Nonbiological	Biological
Celecoxib Etoricoxib Lumiracoxib Parecoxib	Prednisone	Azathioprine Ciclosporin [hydroxy]-chloroquine Leflunomide Methotrexate Sulphasalazine	Anti-TNF-*α* agents: infliximab, adalimumab, etanercept, golimumab, and certolizumab pegol Anti-IL1: anakinra Anti-IL-6: tocilizumab Anti-CD20: rituximab Anti-CD80/86-CD28: abatacept

**Table 2:** Disease-modifying antirheumatic drugs.

Drug	Use	Dosage	Adverse effects	Mechanism
Abatacept	Monotherapy in moderate severe RA or with DMARDs	500/1000 mg iv for 2 weeks then for 4 weeks	Headache, blood hypertension, nausea, infections respiratory system, vertigo, and severe infection	Modulator of T-lymphocyte activation

Adalimumab	Moderate or severe AR	40 mg sc. for 2 weeks	Myositis, headache, rash, nausea, blood hypertension, hyperlipidemia, and higher plasma transaminases levels	Anti-TNF-*α*

Anakinra	Monotherapy in moderate/severe RA in patient with ≥1 DMARD not efficacy	100 mg/die sc.	Abdominal pain, diarrhea, headache, fever, and infections of respiratory system	Anti-IL-1

Certolizumab	Moderate or severe AR	400 mg sc. for 2 weeks then 200 mg for 2 weeks	Rash, headache, blood hypertension, fever, and asthenia	Anti-TNF-*α*

Etanercept	Moderate or severe AR with DMARD	50 mg/weeks sc.	Pain into injection site, infections, headache, rash, vertigo, and asthenia	Anti-TNF-*α*

Golimumab	RA M/S with MTX	50 mg/month sc.	Blood hypertension, higher plasma transaminases levels, and vertigo, rhinitis	Anti-TNF-*α*

Infliximab	Moderate or severe AR with MTX	3 mg/kg iv one time then on 2 and 6 weeks	Fever, headache, rash, myalgia, asthenia, dyspnea, and higher plasma transaminases levels	Anti-TNF-*α*

Rituximab	Moderate or severe AR, with MTX in patient and without response to ≥1 anti-TNF-*α* agent	1000 mg iv in days 1 and 15 then at 24 weeks	Fever, nausea, hypotension, itching, and myelosuppression	Modulator of B-cell activation

**Table 3:** DDIs between MTX and NSAIDs.

Drug	Mechanism	Effects	References
NSAIDs (indomethacin, ketoprofen, naproxen, and diclofenac)	Competition for tubular secretion via the HOAT-3	NSAIDs decrease the excretion of MTX with an increase in ADRs.	[47, 48]
COX-2 inhibitors Etoricoxib	Inhibition of HOAT-3	Decreased excretion of MTX with an increase in ADRs	[49]
Other COX-2 inhibitors (i.e., celecoxib and lumiracoxib)		No data	[50, 51]

**Table 4:** MTX's interaction with DMARDs and immunosuppressant.

Drug	Effects	References
Leflunomide and sulphasalazine	Increase in liver and blood toxicity	[54–62]
	Contrasting data	
Cyclosporine	Renal toxicity	[63]
	No interactions	[64]
Mycophenolate mofetil	No data	

**Table 5:** DDIs between MTX's and biological drugs.

Drug	Effects	References
Infliximab, adalimumab, golimumab, and certolizumab pegol	Increased risk of infections	[70–72]
Etanercept	No DDIs	[72]
Adalimumab	No DDIs	[73]
Tocilizumab, certolizumab pegol, and golimumab	No DDIs	[74–76]
Abatacept	No DDIs	[77]
